# Combination of Acupoints for Alzheimer’s Disease: An Association Rule Analysis

**DOI:** 10.3389/fnins.2022.872392

**Published:** 2022-06-08

**Authors:** Yao-Chin Wang, Chieh-Chen Wu, Abel Po-Hao Huang, Po-Chun Hsieh, Woon-Man Kung

**Affiliations:** ^1^Department of Emergency, Min-Sheng General Hospital, Taoyuan, Taiwan; ^2^Graduate Institute of Injury Prevention and Control, College of Public Health, Taipei Medical University, Taipei, Taiwan; ^3^Department of Healthcare Information and Management, School of Health Technology, Ming Chuan University, Taipei, Taiwan; ^4^Division of Neurosurgery, Department of Surgery, National Taiwan University Hospital, College of Medicine, National Taiwan University, Taipei, Taiwan; ^5^Department of Chinese Medicine, Taipei Tzu Chi Hospital, Buddhist Tzu Chi Medical Foundation, New Taipei City, Taiwan; ^6^Division of Neurosurgery, Department of Surgery, Taipei Tzu Chi Hospital, Buddhist Tzu Chi Medical Foundation, New Taipei City, Taiwan; ^7^Department of Exercise and Health Promotion, College of Kinesiology and Health, Chinese Culture University, Taipei, Taiwan

**Keywords:** association rule analysis, combination of acupoints, Alzheimer’s disease, systematic review, meta-analysis, randomized control trials

## Abstract

**Background:**

Alzheimer’s disease (AD) is an ongoing neurological degeneration characterized by amnesia and a decline in cognitive abilities. Hippocampal neurogenesis is the leading cause of AD. Mild cognitive impairment (MCI), a prodromal state of AD, is mainly due to the degradation of neuropsychiatric manifestations. Previous systematic reviews demonstrated that treatment with acupuncture with Chinese herbs is tolerable and effective in improving cognitive function in patients with AD. Our investigation aimed to discover the main acupoint combination for AD management based on a preceding systematic review and meta-analysis of randomized control trials (RCTs).

**Materials and Methods:**

Our investigation was executed using association rule analysis, which is a common data mining technique accessible within R. Our study elucidated acupoint locations as binary data from 15 of the included studies using the Apriori algorithm.

**Results:**

Thirty-two acupoints were selected from 15 RCTs. The 10 most frequent acupoints were selected. We inspected 503 association rules using the interpreted acupuncture data. The obtained results showed that {SP6, BI10} ≥ {HT7} and {HT7, BI10} ≥ {SP6} were the most associated rules in 15 RCTs.

**Conclusion:**

The combination of acupoints ({SP6, BI10} ≥ {HT7} and {HT7, BI10} ≥ {SP6}) can be acknowledged as a core combination for future acupuncture regimens of AD.

## Introduction

Alzheimer’s disease (AD) is an ongoing neurological degeneration characterized by amnesia and a decline in cognitive abilities (Reddy and Oliver, 2019). These symptoms are the most common etiology of neurogenesis. Mild cognitive impairment (MCI), a prodromal state of AD, is mainly due to the degradation of neuropsychiatric manifestations. Anxiety and restlessness are common side effects in a patient with ADs. Furthermore, side effects, such as urinary problems, intestinal problems, mood disorders, infections, brain injuries, and bony fractures, are the major etiologies of disproportion and unsteadiness in patients with AD (Legg, 2017). Increased life expectancy has not led to a better quality of life among patients with AD, with the occurrence of complications. AD results in a high expenditure burden for social and healthcare systems globally (Luengo-Fernandez et al., 2012). In addition, because of medical complications, patients with AD are attracted to non-pharmacological regimens (Raggi et al., 2017). Therefore, it is crucial to explore complementary treatments for AD.

Acupuncture, an effective treatment method in traditional Chinese medicine, has gained more attention owing to its complementary and alternative consequences in reducing the symptoms of certain illnesses harmlessly and evidently. Acupuncture has recently become a candidate for AD therapy with satisfactory outcomes, in terms of improving cognitive impairment and enhancing memory. On the other hand, improper acupoints choice and selection may induce negative clinical therapy for AD (Chaochao et al., 2018). Indeed, a previous systematic review demonstrated that the treatment of acupuncture with Chinese herbs is tolerable and effective in improving cognitive function in patients with AD (Zhou et al., 2017). Recently, authors discussed the effectiveness and safety of acupuncture for the management of AD in some meta-analysis studies (Huang et al., 2019) (Wang et al., 2020).

Currently, data mining techniques have been extensively adopted in Western and Eastern medicine, such as the discovery of a link between acupoints and sickness to enhance treatment response. A previous study provided an implication to select and combine acupoints for the treatment of vascular dementia in hospital and academic research based on data mining results (Feng et al., 2015). Some insightful recommendations and a selection of acupoint combinations for AD have also been provided (Chaochao et al., 2018). Based on a literature review, data mining has extensively discovered potential acupoints for the efficient management of certain diseases. Researchers have investigated the treatment of primary dysmenorrhea by acupoints through a data mining approach (Yu et al., 2015). Association rule analysis is commonly applied to determine substantial and persistent directional collaborations in marketing between jointly purchased items. Since the scientific process of traditional Chinese medicine and acupuncture is established based on the combination of acupoints or herbs, association rule analysis may become a reliable and helpful approach to determine the essential guidelines of item sets.

Therefore, our investigation aimed to find some possible acupoint combinations to treat AD based on a previous systematic review and meta-analysis of randomized control trials (RCTs) (Zhou et al., 2017).

## Materials and Methods

### Data Sources

Our investigation was executed based on the preceding systematic review and meta-analysis (Zhou et al., 2017). We extracted acupoints by binary data from 15 included studies.

### Inclusion Criteria

As the data were extracted from 15 RCTs, the selection criteria details will not be described here. We summarized three inclusion criteria: clear diagnosis, participants who received acupuncture when they were assigned to the experimental group, and at least one clear outcome stated in the RCTs.

### Data Analysis

First, we extracted data from a previous systematic review and meta-analysis of RCTs. Next, we described acupoints according to the WHO Standard Acupoint Locations in the Western Pacific Region (Lim, 2010). The association rule analysis of the Apriori algorithm (Bayardo and Agrawal, 1999) and mapping in this study were performed using R software (version 3.4.3). This scheme is properly adapted by applying “arules” of the R package. Visualization of association rule can be perfectly matched by applying “arulesViz” of the R package.

The 10 most frequent acupoints were analyzed in our investigation. We set the minimum qualifications as a support degree of ≥20% with confidence of ≥80%. Our study demonstrated an association rule in agreement with descending support. We also reported confidence along with lift values in accordance with the association rule.

## Results

### Distribution of Acupoints

**FIGURE 1:**
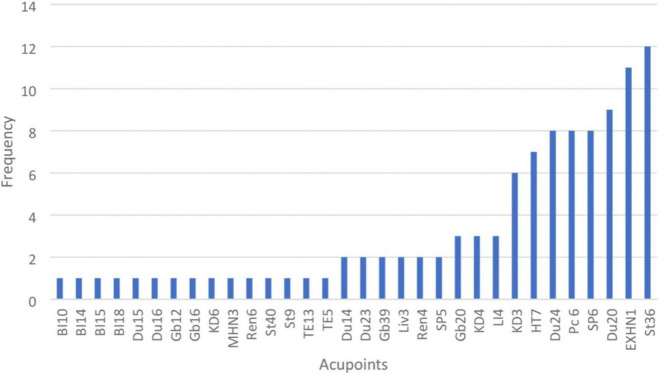
Distribution of acupoints used in the randomized control trials (RCTs).

Thirty-two acupoints were extracted from the 15 RCTs in the aforementioned meta-analysis. The first acupoint is the Tianzhu “Upper Pillar” (BI10), which is located in back of the neck. The 10 most frequently selected acupoints were St36, EXHN1, Du20, SP6, Pc6, Du24, HT7, KD3, LI4, and KD4. The names of each acupoint mentioned above are as follows: Zusanli “Leg Three Miles” (St36), Sishencong “Group of Four Points” (EXHN1), Baihui “One Hundred Meetings” (Du20), Sanyinjiao “Three Yin Intersection” (SP6), Neiguan “Inner Pass” (Pc6), Shenting “Spirit Courtyard” (Du24), Shenmen “Spirit Gate” (HT7), Taixi “Great Stream” (KD3), He Gu “Junction Valley” (LI4), and Dazhong “Large Bell” (KD4). The frequency of acupoints is shown in Figure 1.

### Association Rule Analysis for a Combination of Acupoints

**FIGURE 2:**
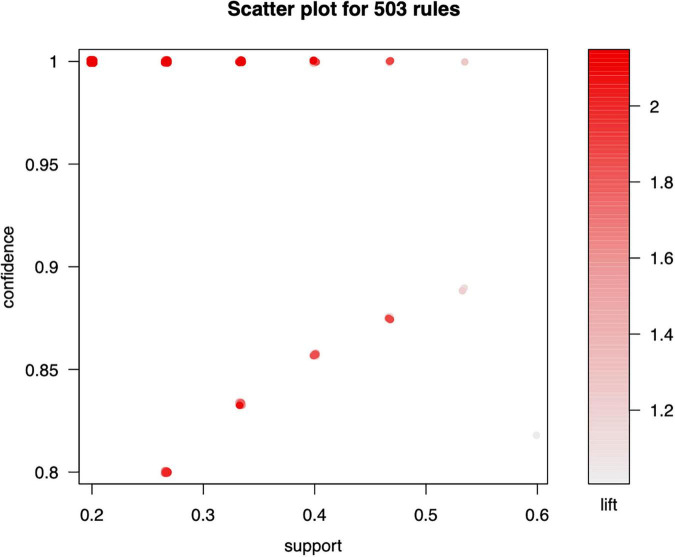
Scatter plot for 503 rules.

We inspected 503 association rules using the interpreted acupuncture data. The scatter plot in Figure 2 visually shows the association rule. The lift value of a rule is the ratio between the observed support and that expected if X and Y are independent. As shown in Figure 2, rules with high lift values obtained comparatively little support. This scatter plot shows the lift on the *y*-axis. The results indicate that all the rules have a high lift value. The most impressive rule (sc-optimal rule) endured on the support/confidence border (Bayardo and Agrawal, 1999) is presented in Figure 2. The association rule between distinct acupoints is ordered by support. The 10 most frequent association rules are presented in Table 1.

**TABLE 1:** Ten most frequent association rules of acupoints.

No.	Association rules	Support	Confidence	Lift
1	{EXHN1} ≥ {St36}	0.6000000	0.8181818	1.022727
2	{SP6} ≥ {St36}	0.5333333	1.0000000	1.250000
3	{Du20} ≥ {EXHN1}	0.5333333	0.8888889	1.212121
4	{Du20} ≥ {St36}	0.5333333	0.8888889	1.111111
5	{HT7} ≥ {SP6}	0.4666667	1.0000000	1.875000
6	{SP6} ≥ {HT7}	0.4666667	0.8750000	1.875000
7	{HT7} ≥ {St36}	0.4666667	1.0000000	1.250000
8	{Pc6} ≥ {Du20}	0.4666667	0.8750000	1.458333
9	{Pc6} ≥ {EXHN1}	0.4666667	0.8750000	1.193182
10	{Pc6} ≥ {St36}	0.4666667	0.8750000	1.093750

**FIGURE 3:**
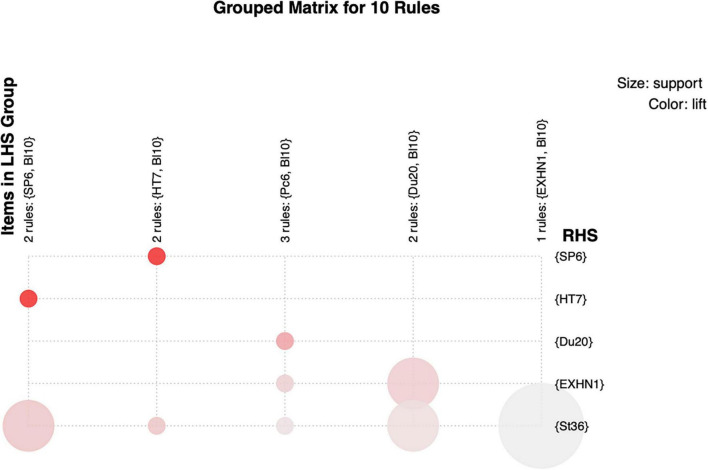
Grouped matrix for 10 association rules.

Our study applied graph-based visualization with pigmentation or magnitude according to the grouped itemsets/rules. Figure 3 shows the characteristic visualization established on a grouped matrix for 10 association rules. It offers a fine depiction of association rules and allows extremely tiny sets of rules to bypass scrambled representations. The outcomes indicated that {SP6, BI10} ≥ {HT7} and {HT7, BI10} ≥ {SP6} were selected collectively to declare the rule’s antecedent (LHS) and consequent (RHS) item sets based on a grouped matrix for 10 association rules. We found that the elected association rules are consistent with rule No. 5: {HT7} ≥ {SP6} and No. 6: {SP6} ≥ {HT7}, in contrast to the results in Table 1.

## Discussion

Our results indicated that {SP6, BI10} ≥ {HT7} and{HT7, BI10} ≥ {SP6} are the core combinations of acupoints for the treatment of AD. According to a previous meta-analysis, this combination of acupoints may be crucial in improving the prognosis of patients with AD. The outcomes may demonstrate further acupuncture point options in treatment strategies.

Several possible mechanisms, by which acupuncture improves AD symptoms, have been reported, including cognitive function, neurobiological activity, and anxiety.

Recently, the effect and mechanism of acupuncture in AD have been confirmed by experimental evidence (Zeng et al., 2013). Acupuncture stimulation improves cholinergic neuronal transmission, trophic factor discharge, and synaptic plasticity. In contrast, it decreases apoptotic/oxidative destruction and reduces amyloid-beta (Aβ) protein levels in the hippocampus and significant brain territories in Alzheimer’s animal models (Zeng et al., 2013). A review of animal-based studies on AD presented the benefits of acupuncture, including supervising glucose metabolism, improving neuronal transmission, decreasing oxidative stress, Aβ protein deposition, and neural apoptosis (Cao et al., 2016).

Currently, preclinical murine studies have been conducted to determine the acupuncture effect and mechanism of AD management. For example, mutations in amyloid precursor protein (APP) in transgenic mice can cause illness (Sturchler-Pierrat et al., 1997). The senescence-accelerated mouse prone (SAMP) phenotype is associated with learning and memory loss (Takeda et al., 1981). For the routine treatment of 15 min daily for 3 months, there is an important improvement to the Lashley III water maze test with electroacupuncture (EA) of GV20 and KI1 in APP transgenic mice with settings of 2 Hz/100 Hz and 3–4 mA (Xue et al., 2009a). Similarly, neuronal mitochondrial destruction in the hippocampal CA1 region was minimized by EA treatment (Xue et al., 2009a). In comparison with sham, the presentation of Aβ and its precursor protein in the cerebral cortex and hippocampal CA1 region are substantially reduced by EA treatment (Xue et al., 2009b). The levels of choline acetyltransferase in these brain regions also increased significantly after EA treatment compared to the control group (Xue et al., 2009b). When compared with the control group, acupuncture stimulation at acupoints GV20 and KI1 may stimulate and raise the presentation of low-density lipoprotein receptor-related protein 1 in the hippocampal sulcus microvessels of APP transgenic mice (Xue et al., 2011).

In SAMP8 mice, stimulants of acupoints CV17, St36, CV12, CV6, and SP10 daily for 15 days showed a significant decrease in discontinuation to discover the hidden platform in the Morris water maze task test in 4-month-old SAMP8 mouse when compared to sham (Cheng et al., 2008). In SAMP10 mice, acupuncture was performed at CV17, CV12, CV6, SP10, and St36 daily for 15 days to investigate the effects of acupuncture on gene expression associated with cerebellar aging in mouse (Ding et al., 2006).

Rats have been a crucial model for AD research for decades. Following sequential research, EA was conducted at acupoints Xiusanzhen, including bilateral LI20 and EXHN3, at 80–100 Hz and 1–3 mA for 10 min, once daily (except weekends) for 1.5 months in rats that acquired intrahippocampal injection of Aβ and established learning and memory deficits (Liu et al., 2009, 2011; Yang et al., 2011). After EA stimulation at acupoints GV20, CV14, KI3, BI23, and St36 once daily for 1 week for one course and lasting for four courses in the Aβ AD rat model, Aβ protein expression was substantially reduced, whereas superoxide dismutases were significantly increased in the hippocampus of acupuncture-treated Aβ rats in comparison with the non-treatment group (Zhang et al., 2010).

From a prospective clinical study, acupuncture stimulants were administered through acupoints HT7 and KI3 for 30 min per course in a 7-day cycle with a 3-day interval between each cycle for a total of 30 days. At the termination of therapy, patients demonstrated noticeable improvement in verbal orientation and motor coordination and had better average mini-mental state examination (MMSE) scores (Kao et al., 2000). To analyze the mechanisms and therapies of AD, 20 patients with AD were treated with acupuncture (Zhu et al., 2010). Acupuncture was performed at acupoints GV20, BI23, SP10, and BI17 in patients with AD for 3 months. Cognitive impairment in patients with AD assessed by the AD Assessment Scale-Cognitive was markedly diminished. It also reduced the levels of iPF2a in the spinal fluid, serum, and urinary samples of patients with AD. The patients were then graded using the MMSE.

Acupuncture remedies not only use one acupoint, but also a combination of acupoints to treat patients with AD. Lu et al. (2017) mentioned that both St36 and HT7 are acupoints commonly used for AD therapy. Researchers have conducted a study involving a multi-acupoint scheme that comprises four different acupoints, such as Siguan (four gates), bilateral Taichong (LR3), and bilateral Hegu (LI4), to treat AD and MCI (Shan et al., 2018). This study successfully demonstrated association rule mining in quantitative RCTs for acupuncture treatment in patients with AD. One study targeted more specific clinical outcomes than mining in a large variety of studies (Yu et al., 2018). Thus, the combination of core acupoints by mining relevant RCTs may provide useful recommendations for more fundamental structural research, clinical trials, and therapeutic approaches.

While we recommend a combination of acupoints, some limitations of this analysis still exist. First, we performed association rule mining on only 15 RCTs without subgroup association rule analysis for specific subgroups of RCTs, such as the duration of the course of treatment. Second, the bias of this study strongly depended on the risk of bias included in the RCTs and publication bias of the included RCTs. Third, the underlying etiology of acupuncture point combinations remains ambiguous. Consequently, further research is required.

## Conclusion

The acupuncture point combination of {SP6, BI10} ≥ {HT7} and {HT7, BI10} ≥ {SP6} can be considered as the main future acupuncture treatment for patients with AD.

## Data Availability Statement

The original contributions presented in this study are included in the article. Further inquiries can be directed to the corresponding author.

## Author Contributions

P-CH and W-MK proposed the research idea, wrote the draft, and contributed to the literature review. Y-CW and P-CH performed the analysis. C-CW and W-MK supported the literature review and helped revise the manuscript. Y-CW and AP-HH provided clinical suggestions. Y-CW and W-MK supported data analysis and prepared the manuscript for submission. All authors read and approved the final manuscript.

## Conflict of Interest

The authors declare that the research was conducted in the absence of any commercial or financial relationships that could be construed as a potential conflict of interest.

## Publisher’s Note

All claims expressed in this article are solely those of the authors and do not necessarily represent those of their affiliated organizations, or those of the publisher, the editors and the reviewers. Any product that may be evaluated in this article, or claim that may be made by its manufacturer, is not guaranteed or endorsed by the publisher.
